# Percutaneous vs. surgical management in acute cholecystitis: addressing selection bias in meta-analyses

**DOI:** 10.1186/s13017-026-00671-5

**Published:** 2026-02-13

**Authors:** Ling Chen

**Affiliations:** Department of Acute Care Surgery, Zhongshan Hospital, Fudan University, Shanghai, 200032 China

**Keywords:** Acute cholecystitis, Percutaneous cholecystostomy, Cholecystectomy, Systematic review, Selection bias, Clinical decision-making

## Abstract

This commentary addresses the recent research titled “Comparing percutaneous treatment and cholecystectomy outcomes in acute cholecystitis patients: a systematic review and meta-analysis” published in the *World Journal of Emergency Surgery*. The review confirms that cholecystectomy (CC), particularly laparoscopic, is associated with lower mortality and readmission rates compared to percutaneous cholecystostomy (PC). However, this commentary emphasizes that the interpretation of these findings must account for inherent selection bias: in clinical practice, PC is typically reserved for higher-risk patients who are unfit for immediate surgery. Thus, the observed outcome differences partially reflect disparities in baseline risk rather than therapeutic efficacy alone. The review’s true value lies in reinforcing early CC as the standard of care for suitable patients and clarifying the role of PC as a “bridge therapy” to stabilize patients for subsequent definitive surgery. Future research should focus on optimizing risk stratification and timing of delayed CC after PC.

## Main text

Dear editor,

I read with interest the systematic review and meta-analysis by Fanciulli and colleagues [1], which provides a quantitative synthesis comparing outcomes between percutaneous cholecystostomy (PC) and cholecystectomy (CC) in patients with acute cholecystitis. The authors are to be commended for undertaking this comprehensive review, which reinforces the established position of early cholecystectomy as the preferred intervention for suitable patients. The purpose of this correspondence is to contextualize and complement these findings by exploring a fundamental challenge in interpreting such comparative studies: the substantial selection bias inherent in their source data, a limitation that warrants careful consideration alongside statistical results.

The meta-analysis demonstrates that patients undergoing CC experience significantly lower mortality and readmission rates than those treated with PC. While this finding is clear, its attribution primarily to the treatment effect is problematic. In clinical reality, the choice between PC and CC is not a random allocation but a deliberate triage based on a composite assessment of risk. The authors correctly note that the included studies focused on high-risk populations, often classified as ASA III or IV. However, the ASA score captures only systemic physiological reserve [2, 3]. Equally, if not more, influential in the surgeon’s decision-making is the local anatomical risk assessed through pre-operative imaging [4, 5]. A patient with an ASA II score but with computed tomography evidence of a phlegmonous mass obscuring the hepatocystic triangle would almost universally be directed towards PC due to the unacceptable risk of iatrogenic bile duct injury. This critical dimension of “technical operability” or “anatomical hostility” is rarely captured in retrospective datasets, yet it systematically enriches the PC cohort with patients facing a higher intrinsic risk of poor outcomes, independent of the treatment modality itself.

Consequently, the compared groups are fundamentally dissimilar at baseline in ways that extend beyond measurable comorbidities. The PC group comprises patients selected for their combined systemic frailty and/or local anatomical complexity, while the CC group consists of patients deemed both systemically and locally suitable for surgery. Therefore, the observed worse outcomes in the PC group are a predictable result of this clinical triage. The meta-analysis effectively compares populations with fundamentally different risk profiles, documenting the consequences of sound clinical judgment rather than providing an unbiased comparison of therapeutic strategies. It answers the question, “Do patients selected for PC do worse than those selected for CC?” with a resounding yes, but it cannot answer the clinically pertinent counterfactual: “Would a specific high-risk patient have fared better with an alternative treatment?”

Given this inherent limitation, the value of this review shifts. Its primary contribution is not in proving the superiority of CC—a conclusion confounded by indication—but in validating the real-world outcomes of distinct clinical pathways and, more importantly, in its nuanced discussion of the staged approach. The data suggesting that PC as a definitive treatment leads to higher rates of recurrent biliary events strengthens the argument against its use as a permanent solution. The most actionable insight is the support for viewing PC as a bridge: a temporizing measure to control sepsis and improve physiological status, with the intent to reassess for delayed cholecystectomy [6, 7]. This aligns perfectly with modern guideline recommendations, emphasizing that the treatment goal should be definitive surgery whenever safely achievable.

In summary, this commentary highlights that interpreting comparative outcomes between PC and CC requires acknowledgment of profound selection bias, particularly related to unmeasured anatomical risk. For clinicians, the key message is clear: percutaneous cholecystostomy is best conceptualized as a bridge to definitive surgery, not as its equivalent alternative. Future studies should focus on optimizing risk stratification and timing of delayed CC after PC. Ultimately, clinical decisions must remain patient-centered, selecting the safest initial path with a view toward achieving curative treatment when possible.

## Data Availability

No datasets were generated or analysed during the current study.

## Abbreviations

AC: Acute cholecystitis
PC: Percutaneous cholecystostomy
CC: Cholecystectomy
ASA: American society of anesthesiologists
